# Data transformation

**DOI:** 10.4103/0976-500X.77115

**Published:** 2011

**Authors:** Jaykaran

**Affiliations:** *Department of Pharmacology, Govt. Medical College, Surat, Gujarat, India*

Sir,

I read with interest the article by Manikanandan S, “Data transformation.”[1] These kinds of articles are very helpful for postgraduates and young researchers. As the article is going to guide postgraduates during analyzing their data of research, I would like to comments on few issues raised in the article:

1. The author mentioned that the reason why the distribution is called normal distribution is that most of the biological variables (weight, height, and blood sugar) follow it. Here I want to emphasize that normal distribution was not discovered for the distribution of biological variables and the reason it is called this is not because most of the biological variables follow it but that it is most frequently seen distribution in nature.
  The statement, “Most biological variable follow the normal distribution,” needs to be analyzed cautiously as opinions are divided between the statisticians and researchers. If we are sure that biological variables like blood pressure, height, and weight always follow the normal distribution then there is no need to check distribution for these variables and parametric tests can be used straightaway but it is observed that distribution is also checked for these variables and nonparametric tests are also used when the distribution is found not following the normal distribution. The distribution of variables also depends on the sample size; when the sample size is small then there are more chances that the distribution becomes nonnormal. Most of the statistical tests are based on the “central limit theorem” and in the case of a small sample size, this theorem loses its validity. My advice for the postgraduates is to check the distribution of their data for all biological variables including weight, height, and blood pressure specially when the sample size is small and subjects are selected nonrandomly. Instead of declaring “most biological variables follow it,” I believe it is better to say that “many biological variables follow it when subjects are selected randomly and the sample size is large.”
2. The author mentioned that “one of the assumptions of statistical tests used for testing hypotheses is that data are sampled from normal distribution;” though the statement is essentially correct, some explanation is needed here to clarify it in an unambiguous way. Here I would like to clarify that fulfillment of this assumption is needed only for parametric statistical tests and not all statistical tests. Fulfillment of this assumption is needed for a “t-test” or “ANOVA” but not for the “Mann–Whitney,” “Kruskal–Wallis,” “chi-square,” or other nonparametric test. Researchers should only think of checking the distribution of data when the data are ratios or intervals, otherwise there is no need to check the distribution in the case of nominal or ordinal data.
3. The author mentioned some simple ways to check skewed distribution. I believe that many other methods could have been incorporated here. Whether the data follow the normal distribution or not can be checked by
  - Plotting a histogram
  - Plotting a box and whisker plot
  - Plotting a Q–Q plot
  - Measuring skewness and kurtosis
  - Using a formal statistical test for normality, etc.
  Out of these methods, a histogram and a box and whisker plot can be plotted easily. Skewness and kurtosis can be measured easily not only by a mathematical formula but also by Excel sheet. There are various statistical tests like Kolmogorov–Smirnov test, Shapiro–Wilk test, D’Agostino–Pearson omnibus test, etc., which can be used to check the distribution of data. I believe that the decision about the distribution of data should be taken after obtaining the results of all methods and also after understanding the distribution of the variable in the population from which the sample was taken.
4. At many places, the author mentioned “skewness” at the place of skewed distribution. Readers should understand that checking skewness (shifting of the curve to left or right) is one component of checking normal distribution as mentioned previously. Skewed distribution is nonnormal distribution.
5. The author mentioned that “once skewness (read “skewed distribution” or “nonnormal distribution”) is identified, every attempt should be made to convert it into normal distribution.” In this case also opinions are divided and some statisticians believe that instead of making various efforts to transform data, a nonparametric test can be applied to these kinds of data.
6. One more component is ignored in this article and that is “conversion of data.” It is observed in various articles published in medical journals that sometimes continuous data are converted into categorical data (ordinal or nominal) by using “cut-off points.” For example, blood pressure (ratio) data can be converted into hypertensive and nonhypertensive (nominal data) or mild hypertension, moderate hypertension, and severe hypertension (ordinal data). This conversion causes cause loss of information, and statistical tests are more sensitive to continuous data than categorical. So if possible during the analysis of data, this conversion should be avoided. If authors want to report such converted data in their research, the reason for doing this conversion should be mentioned, as it causes loss of precision; also how the cut-off boundaries were selected should be mentioned as sometimes cut-off boundaries are selected in such a way that it may favor the results.[2]
